# Methicillin resistant *Staphylococcus aureus* carriage among guinea pigs raised as livestock in Ecuador

**DOI:** 10.1016/j.onehlt.2019.100118

**Published:** 2019-11-29

**Authors:** Marlon Zambrano-Mila, Angel S. Rodriguez, Ismar A. Rivera-Olivero, Mauricio Salas-Rueda, Marco Vinicio Caceres-Orellana, Jacobus H. de Waard, Miguel Angel Garcia-Bereguiain

**Affiliations:** a One Health Research Group, Facultad de Ciencias de la Salud, Universidad de Las Américas, Quito, Ecuador; b Escuela de Ciencias Biológicas e Ingeniería, Universidad Yachay Tech, Urcuquí, Ecuador; c Globalgen, Carrera de Medicina Veterinaria y Zootecnia. Universidad Politécnica Salesiana, Cuenca, Ecuador; d Clínica Veterinaria Cáceres. Paute. Ecuador; e Departamento de Tuberculosis, Instituto de Biomedicina Dr. Jacinto Convit, Universidad Central de Venezuela, Caracas, Venezuela

## Abstract

Antimicrobial resistance is a growing problem in human and veterinary medicine. Here we show that 6.25% of the guinea pigs studied in Ecuador, raised as livestock, carry methicillin resistant *Staphylococcus aureus* (MRSA) in the nasopharynx and therefore may potentially play a role in the transmission of MRSA in the Andean Region of South America.

## Highlights

• Livestock-associated MRSA is a worldwide public health issue.
• Guinea pigs are raised as livestock in the Andean Community, including in Ecuador.
• The presence of MRSA in guinea pigs raised as livestock might be a public health problem that needs further investigation.

## 1 Introduction

*Staphylococcus aureus* (SA) is a human pathogen that causes problems ranging from mild skin and soft tissue infections to severe systemic infections like sepsis and necrotizing pneumonia. The prevalence of SA drug resistant strains is growing worldwide, including methicillin-resistant SA (MRSA) [1,2]. The resistance of MRSA to β-lactam antibiotics is associated with the presence of *mecA* and *mecC* genes, which encode for penicillin-binding proteins. These genes are located on a mobile genetic element called staphylococcal cassette chromosome *mec* (SCCmec) [[1], [2], [3]]. Although MRSA strains were first identified as nosocomial pathogens, in recent years, the majority of MRSA infections are community-acquired (CA-MRSA) and the spread of MRSA strains has become a serious public health problem [1,2]. Moreover, a cytotoxin encoded by *lukS/F-PV* genes that increases SA pathogenicity and virulence is frequently associated with CA-MRSA [4].

The ecological niches of SA strains are the anterior nares, and carriage of *S. aureus* in the nose appears to play a key role in the epidemiology and pathogenesis of infection. SA nasal carriage in the human nasopharynx is highly variable. It is usually from 20 to 30%, but can be higher than 50% for children in developing countries like Ecuador (article under preparation). Prevalence of MRSA among SA in humans is also highly variable- from 6 to 80% in Latin America [5], with one report from Ecuador showing a prevalence of almost 50% of MRSA among SA clinical isolates [6]. There are two studies showing nosocomial acquired MRSA in Ecuador, reporting a nasal carriage rate of 2.5% among intensive care unit workers and 45.9% for medical school students respectively [7,8].

A few of the SA nasal carriage studies in the literature determined that several companion animals may become colonized with MRSA, although the frequency and duration of this colonization is unknown [1,2]. Some of these studies reported a low prevalence of MRSA (0-6%) in dogs and cats and a prevalence of 7% MRSA in horses [2].

Nasal carriage of MRSA has also been reported for livestock. Livestock-associated MRSA (LA-MRSA) has been shown to colonize animals and thus humans with occupational exposure are at a risk of infection. Transmission to humans has been shown in Germany, for instance, where in geographical areas with a comparatively high density of pig farms, LA-MRSA transmitted from livestock accounted for 10% of the MRSA from septicemia and 15% of MRSA from wound infections [9]. Other livestock animals, like cows and chicken, have also been shown to act as an MRSA reservoir [1,2,[9], [10], [11]].

Furthermore, several reports have shown MRSA outbreaks with transmission between pets and owners [1,2]. However, although MRSA clones have been proved to be transmitted from pets to humans, in general, MRSA transmission from humans to animals is considered predominant [1,2].

In the western world, guinea pigs (*Cavia porcellus*), originally native to South America, are kept as laboratory animals or pets. However, in the Andean Community (Colombia, Ecuador, Peru and Bolivia), they are raised for ceremonial purposes or used for meat and are part of the traditional cuisine. They are mass-produced as livestock and/or for self-consumption. They are reared inside people's homes, with up to 50 guinea pigs per household, or in barns for commercial purposes, with up to 3000 animals per barn. In Ecuador, over 700.000 families are in the guinea pig farming business, raising more than 50 million animals per year, but despite its local economical relevance, the production is still traditional and lacks any standards or guidelines on animal health or any sanitary control [12,13].

Outbreaks of zoonotic infections due to pet guinea pigs have been reported, including MRSA carriage [10,11]. Moreover, the presence of zoonotic pathogens like influenza virus or *Campylobacter jejuni* has been shown for livestock guinea pigs [12,13]. However, no researchers have ever investigated farm-raised guinea pigs for MRSA carriage. The aim of this study was to detect and characterize MRSA strains carried in the nose and nasopharynx of guinea pigs raised as livestock in Ecuador.

## 2 Methods

We studied guinea pigs from 6 farms in Azuay, a province of Ecuador located in the Andean region, 2500 m above sea level and one of the main producers of guinea pig meat in Ecuador. We obtained nasopharyngeal samples from 80 guinea pigs by washing the nasopharynx of healthy guinea pigs with a disposable and sterile plastic pipette, carefully pipetting in and out about 2 ml of a sterile saline solution. The samples were aseptically mixed with the same volume of 2× concentrated STGG, a liquid transport medium containing skim milk, tryptone, glucose, and glycerin [14], frozen and transported to the laboratory for the isolation and identification of SA with standard microbiological techniques. Antibiotic susceptibility was performed using the disk-diffusion method, according to Clinical and Laboratory Standard Institute (CLSI) 2019 guidelines. Phenotypically identified MRSA were further genotypically characterized for the presence of *mecA* or *mecC* genes, and also *lukS/F*-PV identification was carried out by single or multiplex PCR [15,16].

## 3 Results

From 24 of the 80 guinea pigs (30%), SA strains were isolated and these were tested for antibiotic susceptibility; 6 of the 24 SA strains (25%) were resistant to cefoxitin and oxacillin and were phenotypically identified as MRSA strains (see Table 1). These strains were further tested for the presence of the *mecA* or *mecC* genes and all six MRSA strains were PCR positive for the *mecA* gene. We also investigated the 24 SA strains for the presence of the gene for the cytotoxin Panton-Valentine leukocidin (PVL). Three SA isolates resulted positive for the PVL gene, including two MRSA strains with the *mecA gene* (see Table 1) and one MRSA strain with the resistance pattern CLIN, TET, CIP and SXT.

**Table 1:** Phenotypic and genotypic characteristics of the MRSA isolates from farm raised Guinea pigs of Ecuador. FOX: cefoxitin; cMLS_B_: resistant to both erythromycin and clindamycin; CLIN: Clindamycin; TET: tetracycline; CIP: ciprofloxacin; SXT: sulfamethoxazole-trimethoprim.

Guinea Pig Code	Antimicrobial resistance		Virulence factors
	***Phenotype***	***Genotype***	***luk-PV***
EV8	FOX- cMLS_B_ -TET-CIP	*mec*A +	−
RR4	FOX-CLIN-TET-CIP-SXT	*mec*A +	−
RR6	FOX- cMLS_B_-TET-CIP	*mec*A +	**+**
RR7	FOX -TET-CIP- SXT	*mec*A +	−
RR13	FOX- CLIN-TET-CIP-SXT	*mec*A +	+
RR15	FOX-cMLS_B_-TET-CIP	*mec*A +	−

## 4 Discussion

To the best of our knowledge, this is the first report of MRSA carriage in guinea pigs raised as livestock. Considering that the Andean Community countries' total population is more than 120 million people, our findings point out a potential new source for CA-MRSA zoonotic transmission that could represent a regionally relevant public health problem. It is important to note that guinea pig farming is strongly associated with the traditionally neglected Andean indigenous population, and poor communities where child stunting, malnutrition and immune-suppression is highly prevalent [13], making them targets for CA pathogens like MRSA. Also, as we have mentioned above, over 700,000 Ecuadorian families benefit from the guinea pig business and the production of guinea pigs is estimated to be over 50 million per year. The discovery of MRSA carriage among these guinea pigs deserves further investigation, as the capacity of LA-MRSA strains to spread has been showed for instance in the Netherlands, where a clone of LA-MRSA first isolated in 2003 from pigs accounted for 40% of the human MRSA isolates by 2010 [1].

Concerning the limitations of this preliminary study, the sample size and the geographical location investigated were limited and should be extended in future surveillance studies to other parts of the Andean region. Furthermore, no risk factors were determined for MRSA carriage and genotypic characterization for the presence of clonal complexes of MRSA and PVL-SA strains must be carried out, especially considering that we found uncommon LA-MRSA strains, all negative for *mecC.* The potential transmission to humans must be addressed in future studies, particularly for the at risk population like guinea pig farmers. Biosafety and hygiene guidelines must be implemented by local animal health authorities to raise awareness about zoonotic disease risks associated with poor hygiene farming management.

## Ethics approval and consent to participate

According to national regulations in Ecuador, the need for ethics approval is unnecessary for sample collection for the diagnosis of farm animals (“Ley Orgánica de Sanidad Agropecuaria” 2017, Asamblea Nacional, República del Ecuador). Written and informed consent was obtained from the farm owners who were informed of the results of this investigation.

## Consent to publish

Written informed consent was obtained from the farms owner who were informed of the results of this investigation and authorized the publication of the data.

## Availability of data and materials

A supplementary file with all the data included in the study is available upon request.

## Funding

None.

## Declaration of Competing Interests

The authors declare that they have no known competing financial interests or personal relationships that could have appeared to influence the work reported in this paper.

## Authors' contributions

All authors contributed towards sample collection and data analysis; JHdW and MAGB composed the manuscript; all authors read and approved the final manuscript.

## References

[1] Filippitzi M.E., Goumperis T., Robinson T., Saegerman C. (2017). Microbiological zoonotic emerging risks, transmitted between livestock animals and humans (2007-2015). Transbound. Emerg. Dis..

[2] Pomba C. (2017). Public health risk of antimicrobial resistance transfer from companion animals. J. Antimicrob. Chemother..

[3] Becker K., Ballhausena B., Köckb R., Kriegeskorte A. (2014). Methicillin resistance in Staphylococcus isolates: the “mec alphabet” with specific consideration of mecC, a mec homolog associated with zoonotic S. aureus lineages. Int. J. Med. Microbiol..

[4] Saeed K. (2018). Panton–valentine leukocidin-positive *Staphylococcus aureus*: a position statement from the International Society of Chemotherapy. Int. J. Antimicrob. Agents.

[5] Guzman-Blanco M., Mejia C., Isturiz R. (2009). Epidemiology of methicillin- resistant Staphylococcus aureus (MRSA) in Latin America. Int. J. Antimicrob. Agents.

[6] Zurita J., Barba P., Ortega-Paredes D., Mora M., Rivadeneira S. (2016). Local circulating clones of *Staphylococcus aureus* in Ecuador. Braz J Infect Dis..

[7] Ruiz A., Mora M., Zurita C., Larco D., Toapanta Y., Zurita J. (2014). Prevalence of methicillin-resistant Staphylococcus aureus among health care workers of intensive care units in Ecuador. J Infect Dev Ctries.

[8] Bastidas C.A., Villacres-Granda I., Navarrete D., Monsalve M., Coral-Almeida M., Cifuentes S.G. (2019). Antibiotic susceptibility profile and prevalence of mecA and lukS-PV/lukF-PV genes in Staphylococcus aureus isolated from nasal and pharyngeal sources of medical students in Ecuador. Infect. Drug Resist..

[9] Cuny C., Wieler L.H., Witte W. (2015). Livestock-associated MRSA: the impact on humans. Antibiotics (Basel)..

[10] Walther B., Wieler L.A., Friedrich A.W., Hanssen A.M., Kohn B., Brunnberg L., Lübke-Becker A. (2008). Methicillin-resistant Staphylococcus aureus (MRSA) isolated from small and exotic animals at a university hospital during routine microbiological examinations. Vet. Microbiol..

[11] Walther B., Wieler L.H., Vincze S., Antão E.M., Brandenburg A., Stamm I., Kopp P.A., Kohn B., Semmler T., Lübke-Becker A. (2012). MRSA variant in companion animals. Emerg. Infect. Dis..

[12] Vasco K., Graham J.P., Trueba G. (2016). Detection of zoonotic enteropathogens in children and domestic animals in a semirural community in Ecuador. Appl. Environ. Microbiol..

[13] Leyva-Grado V.H., Mubareka S., Krammer F., Cárdenas W.B., Palese P. (2012). Influenza virus infection in Guinea pigs raised as livestock, Ecuador. Emerg. Infect. Dis..

[14] Satzke C., Turner P., Virolainen-Julkunen A., Adrian P.V., Antonio M., Hare K.M. (2013). Standard method for detecting upper respiratory carriage of Streptococcus pneumoniae: updated recommendations from the World Health Organization pneumococcal carriage working group. Vaccine.

[15] Zhang K., McClure J.A., Elsayed S., Louie T., Conly J.M. (2008). Novel multiplex PCR assay for simultaneous identification of community-associated methicillin-resistant Staphylococcus aureus strains USA300 and USA400 and detection of *mecA* and Panton-valentine leukocidin genes, with discrimination of Staphylococcus aureus from coagulase-negative staphylococci. J. Clin. Microbiol..

[16] Garcia-Alvarez L. (2011). Meticillin-resistant *Staphylococcus aureus* with a novel mecA homologue in human and bovine populations in the UK and Denmark: a descriptive study. Lancet Infect. Dis..

